# Analysis of Different Lithium Disilicate Ceramics According to Their Composition and Processing Technique—A Systematic Review and Meta-Analysis

**DOI:** 10.3390/ma18122709

**Published:** 2025-06-09

**Authors:** Rubén Guaita-Sáez, Jose María Montiel-Company, Rubén Agustín-Panadero, Carla Fons-Badal, Blanca Serra-Pastor, María Fernanda Solá-Ruiz

**Affiliations:** Department of Dental Medicine, Faculty of Medicine and Dentistry, University of Valencia, C/Gascó Oliag n1, 46010 Valencia, Spain; ruguaisa@alumni.uv.es (R.G.-S.); jose.maria.montiel@uv.es (J.M.M.-C.); rubenagustinpanadero@gmail.com (R.A.-P.); carlafonsbadal@gmail.com (C.F.-B.); m.fernanda.sola@uv.es (M.F.S.-R.)

**Keywords:** lithium disilicate, LDS, zirconia-reinforced lithium silicate (ZLS), advanced lithium disilicate, ALD CAD-CAM, heat pressing, mechanical properties, aesthetic properties

## Abstract

Lithium disilicate ceramics (LDSs) are widely used in restorative dentistry for their excellent aesthetic and mechanical properties. Variants like zirconia-reinforced lithium silicate (ZLS) and advanced lithium disilicate (ALD) were developed to enhance these characteristics. However, differences in their physical and optical properties, as well as the influence of processing techniques (heat pressing vs. CAD-CAM), remain unclear. This study aimed to evaluate the physical and aesthetic properties of LDS, ZLS, and ALD ceramics. A systematic review and meta-analysis following PRISMA guidelines were conducted. Studies published in the last ten years were retrieved from PubMed, Web of Science, Scopus, Cochrane, and Scielo. The inclusion criteria encompassed in vitro studies analyzing LDS, ZLS, and ALD ceramics with quantitative data on mechanical and aesthetic properties. Meta-analyses were performed using a random-effects model, with subgroup analyses based on ceramic type and processing technique. Twenty-two studies met the inclusion criteria. Meta-analyses showed significant differences in flexural strength, hardness, surface roughness, wear, and translucency. The processing technique influenced these properties, with CAD-CAM materials exhibiting distinct performance compared to heat-pressed ceramics. Publication bias was assessed using Egger’s test and the Trim and Fill method, and heterogeneity via meta-regression. LDS showed the highest fracture resistance and least wear, while ALD had greater roughness depth. Heat pressing enhanced hardness and reduced roughness, whereas CAD-CAM improved flexural strength. Considering these findings and study limitations, LDS appears the most suitable option for clinical use due to its superior mechanical performance.

## 1. Introduction

Lithium disilicate ceramics (LDSs) are biphasic materials composed of a vitreous matrix and crystalline phases. This vitreous matrix is mainly composed of silicon dioxide (SiO_2_) and lithium oxide (Li_2_O), although it requires different additives to enhance its chemical and mechanical properties, including aluminum oxide (Al_2_O_3_), potassium oxide (K_2_O), phosphorus oxide (P_2_O_5_), and zirconium oxide (ZrO_2_) [1,2].

This group of ceramics was introduced in the late 1990s under the name IPS-Empress II by Ivoclar Vivadent (Schaan, Liechtenstein), initially used as prosthetic cores through heat pressing. Subsequently, their composition was improved by introducing a higher proportion of lithium disilicate crystals (75%) of larger size (0.5–2.5 microns), launching the IPS e.max Press and IPS e.max CAD range [3].

DSL can be processed using heat pressing or CAD-CAM technology. Heat pressing is a traditional method in which the ceramic is heated in a furnace and pressed at high temperature. In contrast, in CAD-CAM, the restoration is digitally designed and milled from lithium metasilicate blocks, which is the pre-crystallized state of LDS. After milling, the blocks undergo thermal treatment to reach their final state [4].

These ceramics offer great versatility due to their excellent aesthetic and mechanical properties and their high adhesion to dental tissues due to their high silica content. This makes them ideal for various dento-supported prosthetic restorations: conventional crowns, veneers, laminate veneers, inlays, onlays, and overlays [4].

Currently, there are different types of LDS on the market depending on their microstructure or composition, making it important to understand them and review whether differences exist in their properties. Among them, zirconia-reinforced lithium silicate ceramic (ZLS), launched in 2013, combines the advantages of lithium disilicate and zirconia due to the presence of 10% by weight zirconia dissolved in its vitreous matrix. Currently, ZLS is available in a crystallized version, including Celtra Duo (Dentsply Sirona, Charlotte, NC, USA), and a pre-crystallized version, including Vita Suprinity (Vita Zahnfabrik, Bad Säckingen, Germany). Moreover, this group of ceramics can also be processed through heat pressing and CAD-CAM technology [5].

Another recent innovation is advanced lithium disilicate ceramic (ALD), with CEREC Tessera (Dentsply Sirona) being its main reference. This novel ceramic incorporates lithium aluminum silicate crystals called virgilite into its zirconia vitreous matrix. According to the manufacturer, these crystals form during the matrix firing process, giving the structure greater resistance and improved aesthetic qualities. However, this ceramic is only processed through CAD-CAM technology [6].

## 2. Objectives

This systematic review and meta-analysis aim to study the physical and aesthetic properties of three groups of ceramics, namely lithium disilicate ceramics (LDSs), zirconia-reinforced lithium silicate ceramics (ZLSs), and advanced lithium disilicate ceramics (ALDs), to determine whether differences exist between them and whether the processing technique (heat pressing or CAD-CAM technology) influences these properties.

## 3. Materials and Methods

### 3.1. Study Selection and Criteria

Articles were selected following the PRISMA (Preferred Reporting Items for Systematic Reviews and Meta-Analyses) checklist guidelines.

This systematic review was registered on PROSPERO with the registration number CRD420251048670.

A systematic review was conducted based on the following PICO question: which brand of porcelain, considering its composition and processing technique, offers the best properties? Specifically,

- “P” (patients) refers to LDS bars, discs, and crowns.
- “I” (intervention) is the influence of the composition and processing technique on ceramic properties.
- “C” (comparison) includes different ceramic groups (LDS, ZLS, and ALD) and processing techniques available in the market.
- “O” (outcome) comprises the properties of each material: Young’s modulus, flexural strength, fracture resistance, hardness, surface roughness (Ra and Rz), wear, and translucency.

A search was performed using specific keywords combined with Boolean operators (AND and OR) to refine the results.

### 3.2. Search Strategy

A comprehensive search was conducted in databases including PubMed, Web of Science, Scielo, Cochrane, and Scopus. A manual book review was also performed.

The search strategy was independently conducted by three researchers (RG-S, MFS-R, and JMM-C) using combinations of the following keywords: lithium disilicate, lithium silicate, LDS, zirconia, ZLS, advanced lithium disilicate, ALD, properties, composition, resistance, crystallization. Additionally, the commercial names of different ceramic groups, such as IPS e.Max, Vita Suprinity, Tessera, and Celtra, were included.

### 3.3. Inclusion and Exclusion Criteria

The systematic review was performed by two researchers (RG-S and MFS-E), while the meta-analysis was conducted by a blinded investigator (JMM-C).

The studies included met the following criteria (Table 1):

- Published within the last ten years;
- In vitro experimental studies focusing on LDS, ZLS, and ALD ceramics;
- Data presented in numerical form.
- The exclusion criteria included the following:
- In vivo studies;
- Studies analyzing materials other than lithium silicate ceramics;
- Studies with non-numeric or graphical data representation.

### 3.4. Risk of Bias Assessment

The quality of the selected articles was assessed using the modified CONSORT scale for in vitro studies on dental materials. This scale evaluates 14 criteria, including structured abstracts, scientific background, objectives, sample size determination, randomization, blinding, statistical methods, and result precision. The studies were rated based on the number of criteria met.

### 3.5. Meta-Analysis and Meta-Regression Design

Meta-analyses were conducted using the Comprehensive Meta-Analysis V3 software.

- A random-effects model was applied.
- Subgroup analysis was performed (LDS, ZLS, and ALD), with significance set at *p* < 0.05.
- Meta-regressions were used to estimate the moderator effect of ceramic type and processing technique on analyzed variables.

## 4. Results

### 4.1. Study Selection and Description

**Figure 1:**
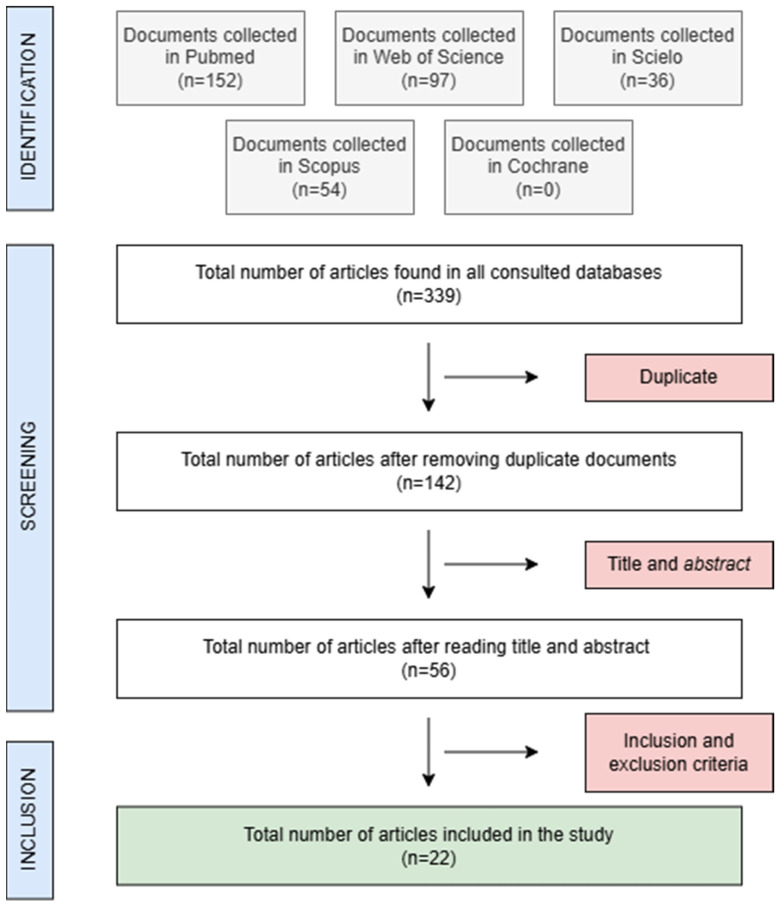
Flowchart of the study selection protocol. PRISMA2009 Flow Diagram.

A total of 437 articles were identified. After refining the search to the last ten years and in vitro studies, 306 remained. After removing duplicates and screening titles and abstracts, 56 articles were selected. Applying inclusion and exclusion criteria further reduced this number to 22. The selection process is detailed in Figure 1. The PRISMA 2020 Flow Diagram [7] can be found in the Supplementary Materials.

The selected articles, including the authors, journal, publication year, and study type, are listed in Table 2. The mechanical properties of each ceramic material, including the Young’s modulus, flexural strength, fracture resistance, Vickers hardness, surface roughness (Ra and Rz), wear, and translucency, are summarized in Table 3.

### 4.2. Methodological Quality

The estimated risk of bias for each included study is presented in Table 4. All studies had structured abstracts, specific objectives, and statistical analyses. However, precision in result estimation and reporting of study limitations varied. The modified CONSORT scale assessment indicated moderate quality, with studies meeting 7 to 9 out of 14 possible criteria.

### 4.3. Results of the Meta-Analysis and Meta-Regression

#### 4.3.1. Young’s Modulus (Stiffness/Elasticity)

**Figure 2:**
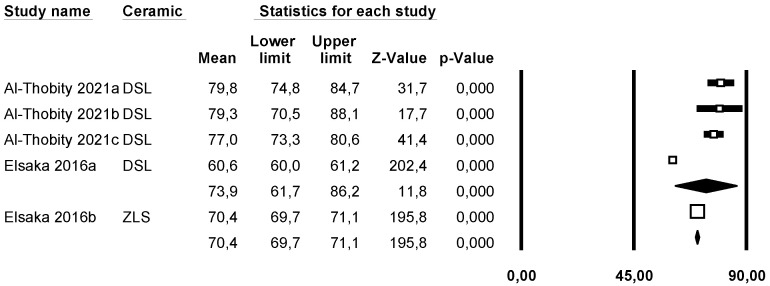
Forest plot: Mean Young’s modulus of the ceramic groups [8,14].

A subgroup meta-analysis combined using a mixed-effects model estimated the effect size for stiffness in the LDS group as 73.9 GPa (95% CI: 61.7–86.2) with an I^2^ = 97.9%. For ZLS, the effect size was 70.4 GPa (95% CI: 69.7–71.1) with an I^2^ = 0%. However, no significant differences were found between the two groups (Q-value 0.31; *p* = 0.577) (Figure 2).

**Figure 3:**
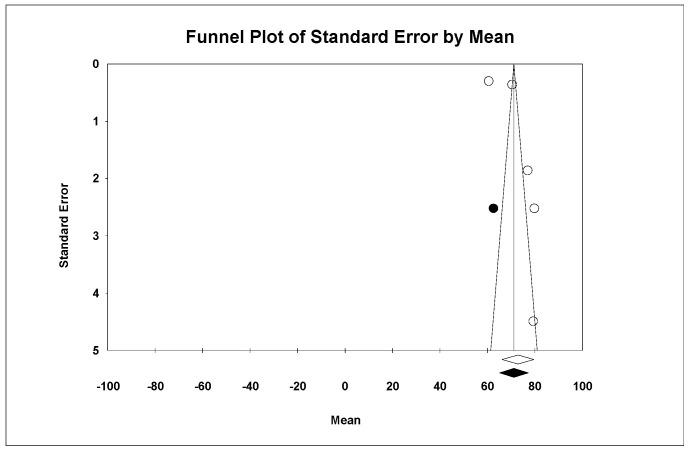
Funnel plot: Standard error by mean of Young’s modulus. For the interpretation of the graph, the white dots represent the studies identified in the systematic review, while the black dots correspond to the new studies imputed using the ‘trim and fill’ method by Tweedie. The diamonds indicate the overall effect estimate.

Using Duval and Tweedie’s Trim and Fill method, one new study was imputed to the left, adjusting the effect size to 71.17 GPa (95% CI: 65.11–72.24), although no significant differences were noted compared to the initial analysis (Figure 3).

Using Egger’s method, the Egger’s regression constant was estimated as 7.1 (95% CI: −17.8 to 32) with a *p* = 0.430.

Based on these two parameters, publication bias was not found.

Through a random-effects meta-regression model, the average Young’s modulus was estimated to be 68.5 GPa (95% CI: 77.5–79.3). With the DSL group as the reference, ZLS would increase the Young’s modulus by 1.9 GPa (*p* = 0.831) and the pressed technique by 9.8 GPa (*p* = 0.203), though neither showed a significant effect. Therefore, the test for this meta-regression model was not significant (Q-value: 1.76; *p* = 0.414; R^2^ = 0%).

#### 4.3.2. Fracture Resistance

**Figure 4:**
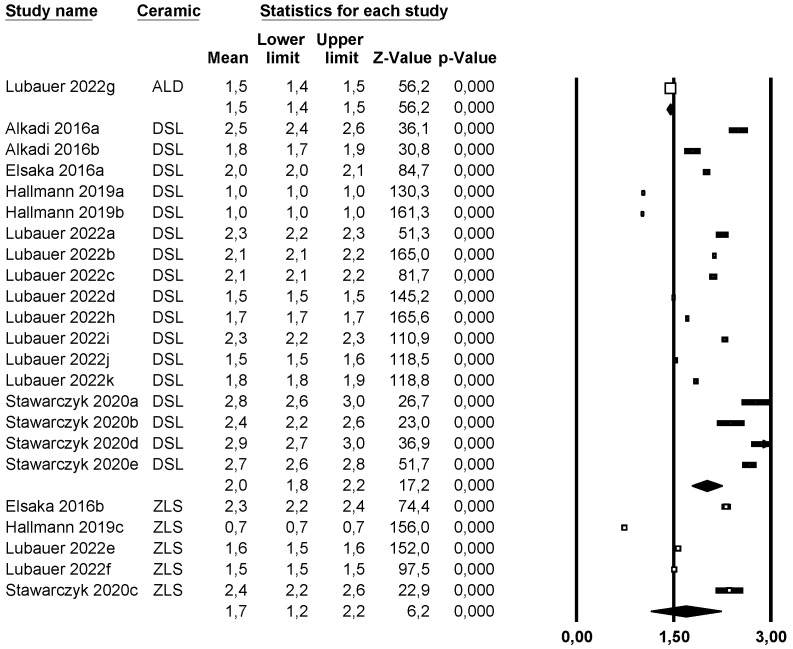
Forest plot: Mean fracture resistance of the ceramic groups [6,9,10,12,14].

A subgroup meta-analysis combined using a mixed-effects model estimated the effect size for fracture resistance in the ALD group as an average of 1.5 MPa·m^1/2^ (95% CI: 1.4–1.5) with an I^2^ = 0%, in LDS as 2.0 MPa·m^1/2^ (95% CI: 1.8–2.2) with an I^2^ = 99.9%, and in ZLS as 1.7 MPa·m^1/2^ (95% CI: 1.2–2.2) with an I^2^ = 99.9%. Significant differences were found between the three groups (Q-value 22.97; *p* < 0.05) (Figure 4).

**Figure 5:**
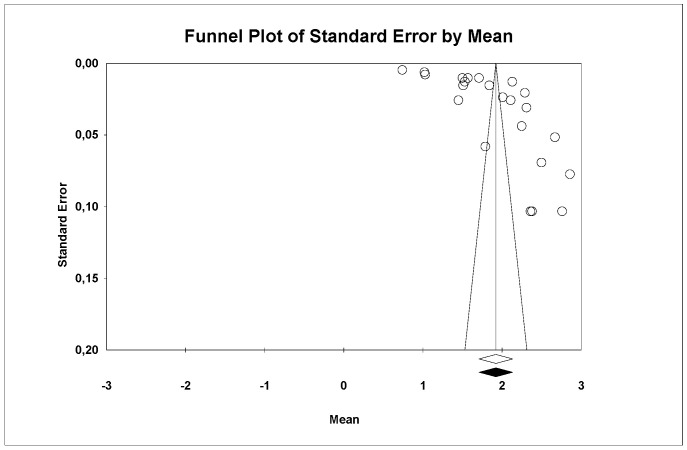
Funnel plot: Standard error by mean of fracture resistance. For the interpretation of the graph, the white dots represent the studies identified in the systematic review. The diamonds indicate the overall effect estimate.

Using Duval and Tweedie’s Trim and Fill method, no new studies were imputed to either side, thus the effect size remained unchanged, and no publication bias was detected (Figure 5).

Using Egger’s method, the Egger’s regression constant was estimated as 41.01 (95% CI: 22.4–59.6) with a *p* < 0.05, indicating publication bias.

Through a random-effects meta-regression model, the average fracture resistance was estimated at 1.9 MPa·m^1/2^ (95% CI: 1.6–2.1). With LDS as the reference group, ALD would decrease fracture resistance by 0.4 MPa·m^1/2^ (*p* = 0.287) and ZLS by 0.3 MPa·m^1/2^ (*p* = 0.150), while the pressed technique would increase it by 0.2 MPa·m^1/2^ (*p* = 0.141), although none of these showed significant effects. The test for this meta-regression model was not significant (Q-value = 6.66; *p* = 0.083; R^2^ = 47%).

#### 4.3.3. Flexural Strength

**Figure 6:**
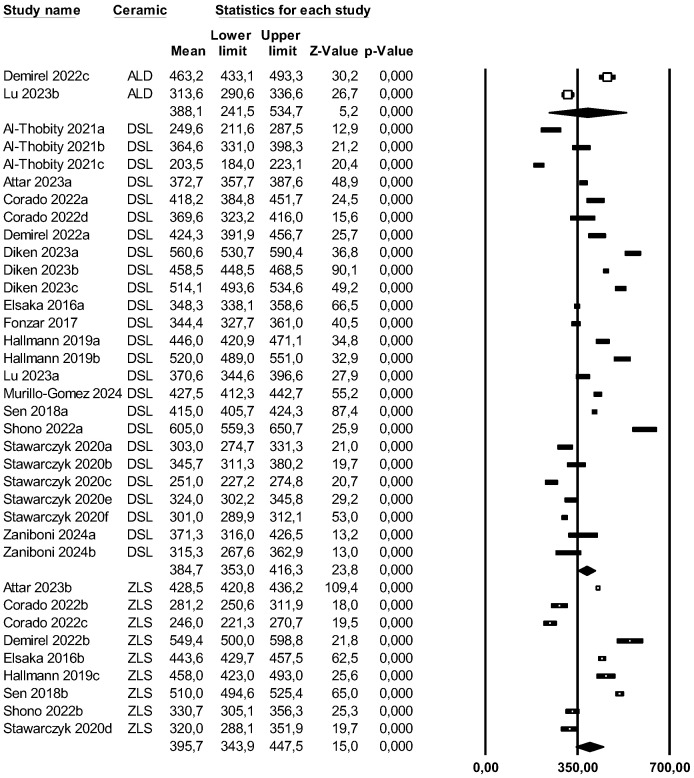
Forest plot: Mean flexural strength of the ceramic groups [8,9,10,13,14,15,18,20,21,23,24,25,27,28].

A subgroup meta-analysis combined using a mixed-effects model estimated the effect size for flexural strength in the ALD group as an average of 388.1 MPa (95% CI: 241.5–534.7) with an I^2^ = 98.3%, in LDS as 384.7 MPa (95% CI: 353.0–416.3) with an I^2^ = 98.5%, and in ZLS as 395.7 MPa (95% CI: 343.9–444.5) with an I^2^ = 98.5%. No significant differences were found between the three groups (Q-value = 0.126; *p* = 0.939) (Figure 6).

Using Egger’s method, the Egger’s regression constant was estimated as −2.02 (95% CI: −7.69 to 3.64) with a *p* = 0.472.

Based on the results obtained from Egger’s method and the fact that Duval and Tweedie’s Trim and Fill imputed a study to the right but none to the left, publication bias was excluded.

Through a random-effects meta-regression model, the average flexural strength was estimated at 411.5 MPa (95% CI: 377.8–445.3). With LDS as the reference group, ALD would decrease flexural strength by 23.8 MPa (*p* = 0.653) and ZLS would increase it by 0.2 MPa (*p* = 0.995). Neither showed a significant effect, but the pressed technique would decrease it by 73.8 MPa (*p* < 0.05), showing a significant effect. Therefore, the test for this meta-regression model was significant (Q-value = 7.99; *p* = 0.046; R^2^ = 19%).

#### 4.3.4. Hardness

**Figure 7:**
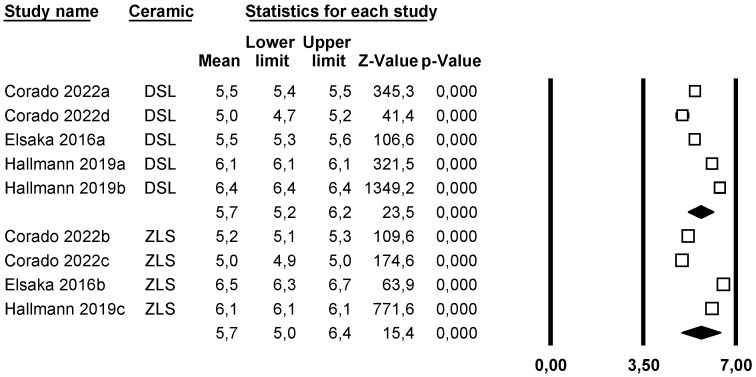
Forest plot: Mean hardness of the ceramic groups [9,14,20].

A subgroup meta-analysis combined using a mixed-effects model estimated the effect size for hardness in the LDS group as 5.7 GPa (95% CI: 5.2–6.2) with an I^2^ = 99.9%, and for ZLS as 5.7 GPa (95% CI: 5.0–6.4) with an I^2^ = 98.7%. No significant differences were found between groups (Q-value 0.001; *p* = 0.979) (Figure 7).

**Figure 8:**
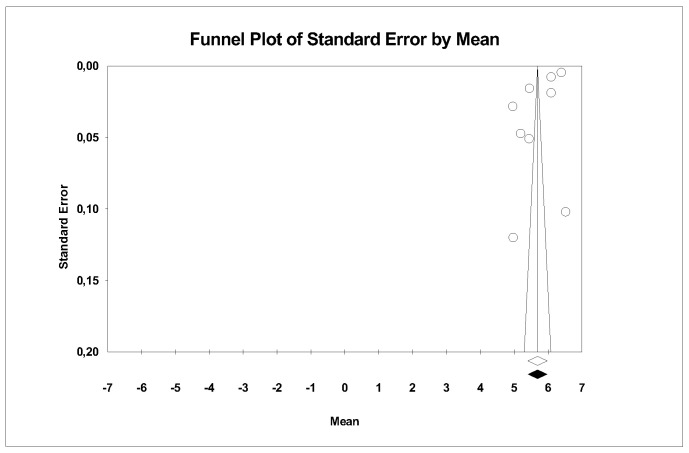
Funnel plot: Standard error by mean of hardness. For the interpretation of the graph, the white dots represent the studies identified in the systematic review. The diamonds indicate the overall effect estimate.

Using Duval and Tweedie’s Trim and Fill method, no new studies were imputed to either side, so the effect size remained unchanged and no publication bias was detected (Figure 8).

Using Egger’s method, the Egger’s regression constant was estimated as −26.06 (95% CI: −50.71 to −1.41) with a *p* = 0.04, indicating publication bias.

Through a random-effects meta-regression model, the average hardness was estimated at 5.4 GPa (95% CI: 5.1–5.6). With LDS as the reference group, ZLS would increase hardness by 0.1 GPa (*p* = 0.604), but the pressed technique would increase it by 0.8 GPa (*p* < 0.05), showing a significant effect. The test for this meta-regression model was significant (Q-value = 26.96; *p* < 0.05; R^2^ = 76%).

##### Roughness (Ra) (Average Roughness)

**Figure 9:**
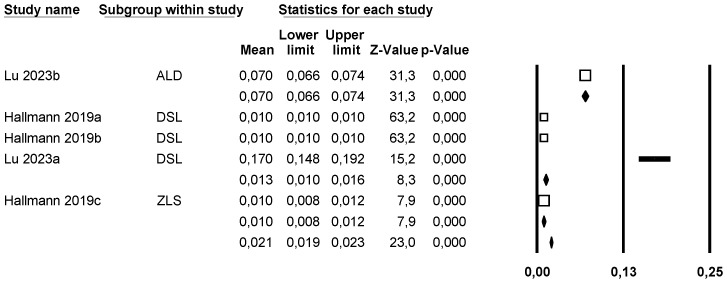
Forest plot: Mean average roughness (Ra) of the ceramic groups [9,23].

Conducting a subgroup meta-analysis combined using a mixed-effects model, the effect size of roughness (Ra) was estimated for the ALD group at 0.07 µm (95% CI 0.066–0.074) with I^2^ = 0%, for LDS at 0.013 µm (95% CI 0.01–0.016) with I^2^ = 99%, and for ZLS at 0.01 µm (95% CI 0.008–0.012) with I^2^ = 0%. Therefore, significant differences between groups were observed (Q-value 2; *p* < 0.05) (Figure 9). 

**Figure 10:**
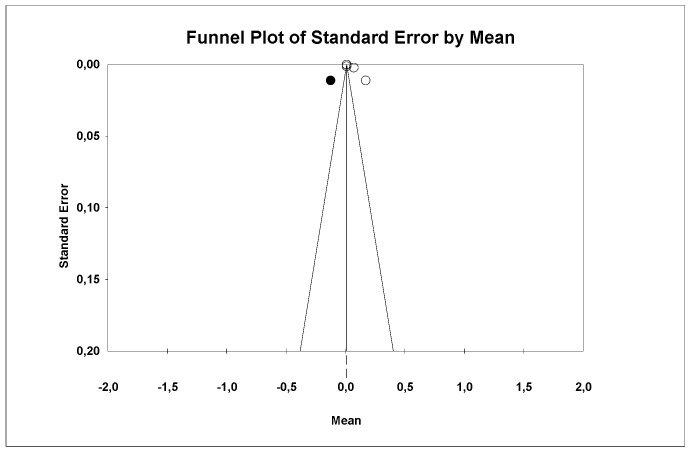
Funnel plot: Standard error by mean of average roughness (Ra). For the interpretation of the graph, the white dots represent the studies identified in the systematic review while the black dots correspond to the new studies imputed using the ‘trim and fill’ method by Tweedie.

Using Duval and Tweedie’s Trim and Fill method, one new study was imputed to the left, slightly modifying the effect size to 0.01 (95% CI 0.009–0.01) without significant differences, and therefore, we discard a publication bias (Figure 10).

Using Egger’s method, it was estimated that the intercept of Egger’s regression line is equal to 15.03 (95% CI −5.99–36.06) with a *p* = 0.10, indicating that there is no publication bias.

Through a random-effects meta-regression model, the mean roughness (Ra) was estimated at 0.013 µm (95% CI 0.01–0.016). With LDS as the reference group, ZLS would decrease the mean roughness by 0.003 µm (*p* = 0.31); however, ALD would increase it by 0.05 µm, showing a significant effect (*p* < 0.05). Therefore, the test for this meta-regression model is significant (Q-value = 281.79; *p* < 0.05; R^2^ = 77%).

##### Roughness (Rz) (Mean Roughness Depth)

**Figure 11:**
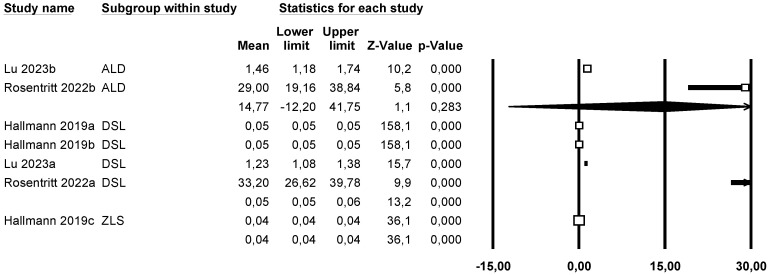
Forest plot: Mean roughness depth (Rz) of the ceramic groups [9,17,23].

A subgroup meta-analysis combined using a mixed-effects model estimated the effect size for roughness (Rz) in the ALD group as 14.8 µm (95% CI: −12.2 to 41.7) with an I^2^ = 96.7%, in LDS as 0.05 µm (95% CI: 0.05 to 0.06) with an I^2^ = 99.1%, and in ZLS as 0.04 µm (95% CI: 0.038 to 0.042) with an I^2^ = 0%. Significant differences were found between groups (Figure 11).

**Figure 12:**
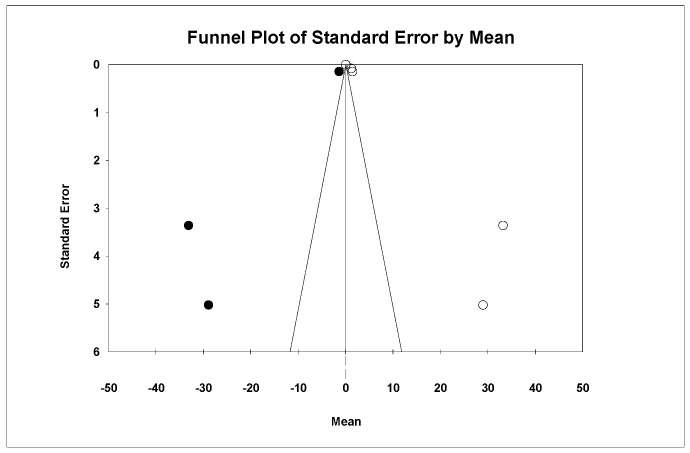
Funnel plot: Standard error by mean of roughness depth (Rz). For the interpretation of the graph, the white dots represent the studies identified in the systematic review while the black dots correspond to the new studies imputed using the ‘trim and fill’ method by Tweedie.

Using Duval and Tweedie’s Trim and Fill method, three new studies were imputed to the left, slightly modifying the effect size to 0.049 (95% CI 0.049–0.05), with no significant differences, thus ruling out publication bias (Figure 12).

Using Egger’s method, the constant of the Egger’s line was estimated to be 7.65 (95% CI −1.28–16.60), with a *p* = 0.079, indicating no publication bias.

Through a random effects meta-regression model, an average Rz of 1.24 µm (95% CI 1.1–1.4) was estimated. With LDS as the reference group, ALD increased roughness by 0.2 µm, while ZLS decreased it by 0.01 µm (*p* < 0.05), and the pressed technique increased it by 1.2 µm (*p* < 0.05), showing a significant effect for these two variables. The test of this meta-regression model is significant (Q-value = 340.62; *p* < 0.05; R^2^ = 75%).

#### 4.3.5. Wear of the Restoration

**Figure 13:**
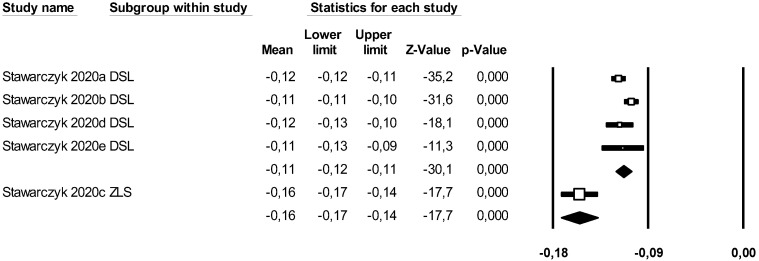
Forest plot: Mean wear of the ceramic groups [10].

A subgroup meta-analysis combined using a mixed-effects model estimated the effect size of wear for the LDS group as −0.11 mm^3^ (95% CI −0.12–(−0.10)) with an I^2^ = 56.75%. For ZLS, the effect size was −0.15 mm^3^ (95% CI −0.17–(−0.13)) with an I^2^ = 0%. Significant differences were found between groups (Q-value 19.15; *p* < 0.05) (Figure 13).

**Figure 14:**
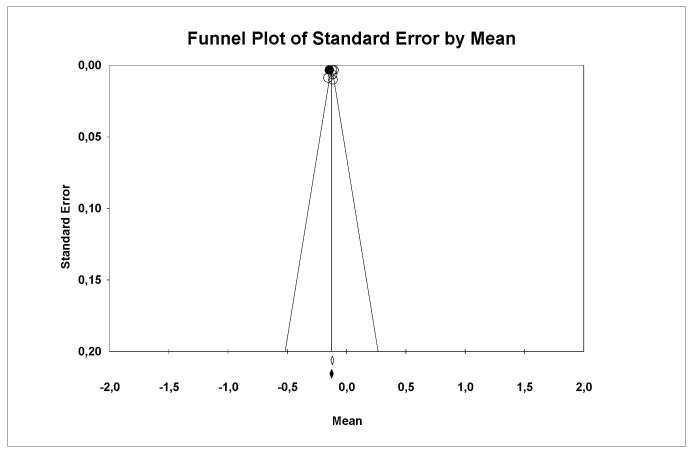
Funnel plot: Standard error by mean of wear. For the interpretation of the graph, the white dots represent the studies identified in the systematic review while the black dots correspond to the new studies imputed using the ‘trim and fill’ method by Tweedie.

Using Duval and Tweedie’s Trim and Fill method, one new study was imputed to the left, slightly modifying the effect size to −0.12 (95% CI −0.14–(−0.11)), with no significant differences, ruling out publication bias (Figure 14).

Using Egger’s method, the constant of the Egger’s line was estimated to be −3.34 (95% CI −12.04–5.34), with a *p* = 0.307, indicating no publication bias.

Through a random effects meta-regression model, an average wear of −0.11 mm^3^ (95% CI −0.12–(−0.10)) was estimated. With LDS as the reference group, ZLS reduced wear by 0.04 mm^3^ (*p* < 0.05), showing a significant effect. Therefore, the test of this meta-regression model is significant (Q-value = 14.48; *p* < 0.05; R^2^ = 82%).

#### 4.3.6. Translucency

**Figure 15:**
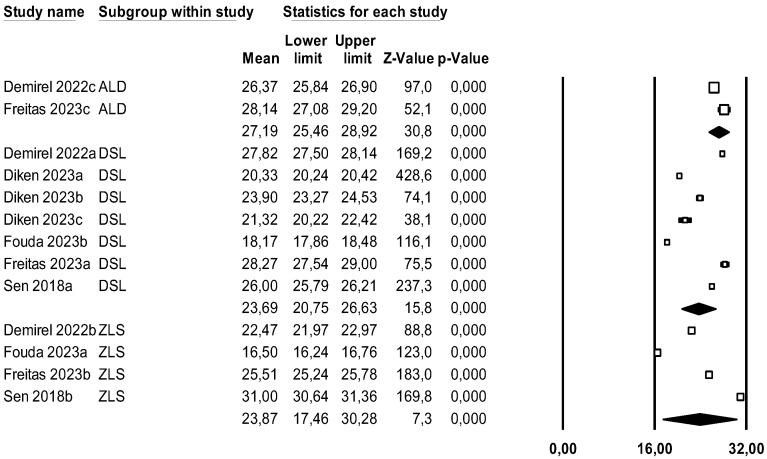
Forest plot: Mean translucency of the ceramic groups [15,18,22,25,26].

A subgroup meta-analysis combined using a mixed-effects model estimated the effect size for translucency for the ALD group as an average of 27.2% (95% CI 25.4–28.1) with an I^2^ = 88.3%, for LDS 23.7% (95% CI 20.7–26.6) with an I^2^ = 99.9%, and for ZLS 23.9% (95% CI 17.4–30.3) with an I^2^ = 99.9%. No significant differences were found between groups (Q-value = 4.576; *p* = 0.101) (Figure 15).

**Figure 16:**
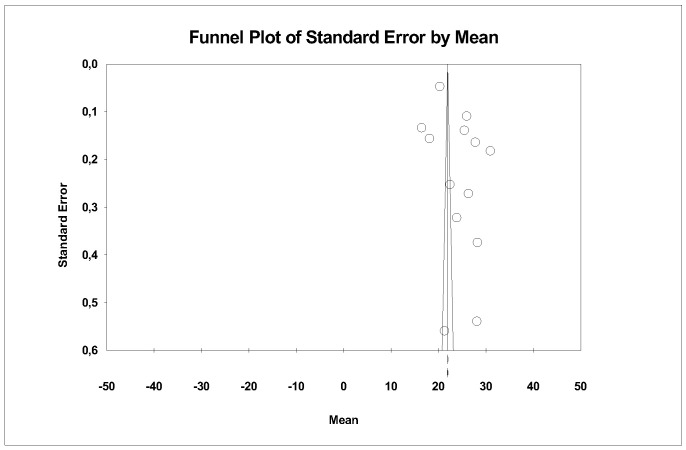
Funnel plot: Standard error by mean of translucency.

Using Duval and Tweedie’s Trim and Fill method, no new studies were imputed to either the left or right, meaning the effect size remains unchanged and thus ruling out publication bias (Figure 16).

Using Egger’s method, the constant of the Egger’s line was estimated to be 20.48 (95% CI −5.73–46.69), with a *p* = 0.113, indicating no publication bias.

Through a random effects meta-regression model, an average translucency of 23.7% (95% CI 20.1–27.2) was estimated. With LDS as the reference group, ZLS increased translucency by 3.6% (*p* = 0.351), and the pressed technique increased translucency by 0.2% (*p* = 0.951), showing no significant effect. The test of this meta-regression model is not significant (Q-value = 0.91; *p* = 0.634; R^2^= 0%).

## 5. Discussion

Once the results were analyzed, the different materials were compared against each other based on the values obtained for each parameter (Table 5).

### 5.1. Young’s Modulus (Stiffness/Elasticity)

In our study, no significant differences in rigidity or elasticity were found between the ceramic groups or processing techniques. It is worth noting that this property could only be studied for the LDS and ZLS groups by two authors (two and one authors, respectively), with no data available for the ALD group.

Al-Thobity, A.M., et al., 2021 [8] corroborates our results, as in our study, it was observed that the LDS ceramics exhibited slightly higher rigidity than the ZLS ceramics, with no significant differences between groups. In contrast, the study by Elsaka, S.E., et al., 2016 [14] observed a higher elastic modulus in the ZLS ceramic (Vita Suprinity), which was statistically significant.

### 5.2. Fracture Resistance

Fracture resistance was studied in all three ceramic groups; specifically, LDS was analyzed by five authors [6,9,10,12,14], ZLS by four authors [6,9,10,14], and ALD by one author [6].

The meta-analysis showed that the LDS group exhibited the highest fracture resistance, followed by ZLS and ALD, with significant differences between them (Q-value 22.97; *p* < 0.05). These results were corroborated by Salem, B.O., et al., 2022 [11], who compared the fracture resistance of LDS versus ZLS, finding that the LDS ceramic (IPS e.max Press) presented higher values than the ZLS ceramic (Celtra Press, York, PA, USA). However, studies by Elsaka, S.E., et al., 2016 [14] and Hamza, T.A., et al., 2019 [19] reported higher fracture resistance in ZLS ceramics (Vita Suprinity), with statistically significant differences observed in both studies.

On the other hand, no significant differences were found regarding the processing technique, although authors like Lubauer, J., et al., 2021 [6] observed higher fracture resistance in thermally pressed ceramics.

Therefore, it can be concluded that the LDS group exhibited the highest fracture resistance among the three groups and that the processing technique did not influence the results.

### 5.3. Flexural Strength

Flexural strength was the most studied property in our systematic review and meta-analysis. Specifically, LDS was studied by 14 authors [8,9,10,13,14,15,18,20,21,24,25,26,27,28], ZLS by 8 authors [9,10,14,15,18,20,21,25], and ALD by 2 authors [18,24].

In our results, no statistically significant differences were observed between the three ceramic types, although slightly higher flexural strength values were observed in ZLS, followed by the ALD and LDS groups. However, the study by Corado, H.P.R., et al., 2022 [20], which compared the properties of various ceramics, observed that the ceramic with the highest flexural strength was LDS (IPS e.max CAD, Ivoclar Vivadent).

In our meta-regression model, it is confirmed that the processing technique had a significant effect, as pressed ceramics showed considerably lower values than CAD-CAM ceramics (Q-value = 7.99; *p* = 0.046; R^2^ = 19%). Authors like Al-Thobity, A.M., et al., 2021 [8] support our results, as they observed that pressed ceramics exhibited lower flexural strength than CAD-CAM ceramics.

In contrast, some studies found no significant differences between both processing techniques (Fabian Fonzar, R., et al., 2017 [13]).

Therefore, it can be observed that the flexural strength of the three groups is similar, and the processing technique does influence the results, with superior performance seen with CAM technology.

### 5.4. Hardness

This property could only be studied in the LDS and ZLS ceramics, with the same authors studying both groups [9,14,20].

In our results, we observed that the average hardness of both groups was similar, with an average hardness for LDS estimated at 5.4 GPa (95% CI 5.1–5.6), and a slightly higher value for ZLS, with a 0.1 MPa difference (*p* = 0.604), showing no statistically significant differences.

Upon conducting the meta-regression, it was observed that the pressed technique increased hardness by 0.8 GPa (*p* < 0.05), showing a significant effect. The test for this meta-regression model is significant (Q-value= 26.96; *p* < 0.05; R^2^ = 76%).

As seen in Table 3, the three ceramics with the highest hardness were processed by thermal pressing, while the rest used CAD-CAM technology. This confirms that ceramics processed by heat are harder than CAD-CAM ceramics. These results are reasonable, as CAM machines require softer materials to perform optimal carving.

Therefore, it can be deduced that the hardness is the same in both the LDS and ZLS groups, and the processing technique does influence the results, being superior with the pressed technology.

### 5.5. Roughness (Ra) (Mean Roughness)

This property was studied in all three ceramic groups; specifically, LDS was studied by three authors [9,17,24], ZLS by one [9], and ALD by two [17,24], with no significant differences between groups.

However, the meta-regression model demonstrated that pressed ceramics exhibited less roughness compared to those processed with CAD-CAM technology, showing a significant effect (Q-value = 522.67; *p* < 0.05; R^2^ = 84%). This could be due to the larger crystal sizes in CAD-CAM ceramics compared to those in pressed ceramics, resulting in higher roughness and consequently worse aging of restorations due to greater plaque retention.

Therefore, it can be concluded that the mean roughness is similar for the three ceramic groups, but the processing technique does have a significant effect, as it decreases Ra in thermally pressed ceramics.

#### Roughness (Rz) (Mean Depth of Roughness)

This parameter was also studied in all three ceramic groups. In the DSL group, it was analyzed by three authors [13,21,28], ZLS by one [9], and ALD by two [17,24].

It was observed that the ALD group showed much higher values compared to the LDS and ZLS groups, with significant differences between them (Q-value = 11.102; *p* < 0.05). Additionally, as seen with the mean roughness (Ra), it is worth noting that the pressed technique had a significant effect on ceramics, as it reduced their roughness (Q-value = 340.62; *p* < 0.05; R^2^ = 75%).

Therefore, it can be deduced that the depth of mean roughness (Rz) is greater in ALD, and the processing technique does influence it, being lower with the pressed technology [29,30].

### 5.6. Wear

In our results, we observed that the LDS ceramic group showed less wear compared to ZLS, with a significant difference between them (Q-value = 19.15; *p* < 0.05). This property was studied exclusively for the LDS and ZLS groups in the article by Stawarcyk, B., et al., 2020 [10]. Furthermore, it is worth noting that all the ceramics in which this parameter was studied were processed by thermal pressing. These results were interesting as the pressed ceramic group exhibited less surface wear and showed the highest hardness, demonstrating the relationship between these two mechanical properties.

Therefore, it can be concluded that for thermally pressed ceramics, LDS exhibits less wear than ZLS.

### 5.7. Translucency

This parameter was studied in all three ceramic groups; specifically, LDS was studied by five authors [15,18,23,26,27], ZLS by four [15,18,23,27], and ALD by two authors [18,27].

In our results, no significant differences were observed between ceramic groups, although higher values were observed in the ALD group. The study by Freitas, J.S., et al., 2023 [26] observed that ALD ceramics (CEREC Tessera) exhibited translucency similar to LDS ceramics (IPS e.max CAD) but superior to ZLS (Vita Suprinity). On the other hand, the article by Fouda, A.M., et al., 2023 [22], comparing the translucency of two LDS ceramics (IPS e.max CAD and LiSi Block) versus a ZLS (Celtra Duo), found that one of the LDS ceramics (LiSi Press) exhibited statistically superior translucency, while the other two porcelains showed similar results.

In our results, no significant differences were observed between ceramic groups or processing techniques.

On the other hand, it is important to comment on the limitations of this study. First, some of the materials included in this review and meta-analysis were studied in a very small number of articles (some by a single author), while other more widely known and used ceramics were studied by numerous authors. Therefore, to recommend them for clinical use, it would be valuable for these materials to be included in more studies.

Second, there were articles with very small sample sizes (between 5 and 15 specimens), while other authors used samples of up to 60 specimens, providing more robust results. Lastly, the quality of all articles was quite similar, as they met between 8 and 9 out of 14 possible items, indicating a moderate quality.

### 5.8. Clinical Relevance

This research holds significant clinical relevance as it focuses on ceramic materials widely used in minimally invasive dental restorations, such as veneers, inlays, onlays, and thin crowns [31,32,33,34]. These treatment approaches aim to preserve as much natural tooth structure as possible, which has been shown to contribute to better long-term prognosis for tooth survival and overall oral health [35,36]. By evaluating the mechanical and aesthetic properties of lithium disilicate-based ceramics, our study helps identify the most suitable materials for such conservative procedures. The use of durable, high-performance ceramics enhances the functional longevity of restorations [37,38], ensuring optimal masticatory efficiency—a factor closely linked to improved quality of life, particularly in aging populations and patients with compromised dentition [3,39]. Our findings provide clinicians with evidence-based guidance to make informed decisions that align with biological, functional, and aesthetic goals in restorative dentistry.

## 6. Conclusions

Finally, after analyzing and discussing the results obtained and considering the limitations of the study, the following conclusions were drawn:

1. The LDS presented the highest fracture resistance of the three groups studied (LDS, ZLS, and ALD) and the least wear. No significant differences were found in flexural strength, hardness, stiffness, translucency, or mean roughness; however, the roughness depth was greater in ALD.
2. The processing technique in the dental laboratory does influence the properties of the ceramics. The press technique increases hardness and decreases roughness, while the CAD-CAM technique showed better results in flexural strength.
3. The most suitable ceramics for clinical use, based on the results obtained and considering the limitations of the study, would be those of LDS due to their physical properties, particularly their high fracture resistance.

## Figures and Tables

**Table 1:** Inclusion and exclusion criteria.

Inclusion Criteria	Exclusion Criteria
In vitro experimental studies	In vivo experimental studies
Lithium disilicate	Materials other than lithium disilicate
Numerical data	Lack of numerical data or data presented in graphs

**Table 2:** General data of the selected articles.

Nº	Author	Year	Title	Journal	Type of Study
***1*** [6]	Lubauer, J., Belli, R., Peterlik, H., Hurle, K., Lohbauer, U.	2022	Grasping the Lithium hype: Insights into modern dental Lithium Silicate glass-ceramics.	Dental Materials	In vitro study
***2*** [8]	Al-Thobity, A.M., Alsalman, A.	2021	Flexural properties of three lithium disilicate materials: An in vitro evaluation.	The Saudi dental journal	In vitro study
***3*** [9]	Hallmann, L., Ulmer, P., Gerngross, M., Jetter, J., Mintrone, M., Lehmann, F.	2019	Properties of hot-pressed lithium silicate glass-ceramics.	Dental Materials	In vitro study
***4*** [10]	Stawarczyk, B., Dinse, L., Eichberger, M., Jungbauer, R., Liebermann, A.	2020	Flexural strength, fracture toughness, three-body wear, and Martens parameters of pressable lithium-X-silicate ceramics.	Dental Materials	In vitro study
***5*** [11]	Salem, B.O., Elshehawi, D.M., Elnaggar, G.A.	2022	Fracture resistance of pressed ZLS crowns versus pressed LD crowns under thermo-mechanical cycling.	Brazilian Dental Journal	In vitro study
***6*** [12]	Alkadi, L., Ruse, N.D.	2016	Fracture toughness of two lithium disilicate dental glass ceramics.	The Journal of prosthetic dentistry	In vitro study
***7*** [13]	Fabian Fonzar, R., Carrabba, M., Sedda, M., Ferrari, M., Goracci, C., Vichi, A.	2017	Flexural resistance of heat-pressed and CAD-CAM lithium disilicate with different translucencies.	Dental Materials	In vitro study
***8*** [14]	Elsaka, S.E., Elnaghy, A.M.	2016	Mechanical properties of zirconia reinforced lithium silicate glass-ceramic.	Dental Materials	In vitro study
***9*** [15]	Sen, N., Us, Y.O.	2018	Mechanical and optical properties of monolithic CAD-CAM restorative materials.	The Journal of prosthetic dentistry	In vitro study
***10*** [16]	Sieper, K., Wille, S., Kern, M.	2017	Fracture strength of lithium disilicate crowns compared to polymer-infiltrated ceramic-network and zirconia reinforced lithium silicate crowns.	Journal of the mechanical behavior of biomedical materials	In vitro study
***11*** [17]	Rosentritt, M., Schmid, A., Huber, C., Strasser, T.	2022	In Vitro Mastication Simulation and Wear Test of Virgilite and Advanced Lithium Disilicate Ceramics.	The International journal of prosthodontics	In vitro study
***12*** [18]	Demirel, M., Diken Türksayar, A.A., Donmez, M.B.	2022	Translucency, color stability, and biaxial flexural strength of advanced lithium disilicate ceramic after coffee thermocycling.	Journal of Esthetic and Restorative Dentistry	In vitro study
***13*** [19]	Hamza, T.A., Sherif, R.M.	2019	Fracture Resistance of Monolithic Glass-Ceramics Versus Bilayered Zirconia-Based Restorations.	Journal of Prosthodontics	In vitro study
***14*** [20]	Corado, H.P.R., da Silveira, P.H.P.M., Ortega, V.L., Ramos, G.G., Elias, C.N.	2022	Flexural Strength of Vitreous Ceramics Based on Lithium Disilicate and Lithium Silicate Reinforced with Zirconia for CAD/CAM.	International Journal of Biomaterials	In vitro study
***15*** [21]	Shono, N., Elhejazi, A., Maawadh, A., Al Nahedh, H.	2022	Ball-on-three-balls biaxial flexural strength of bonded and unbonded CAD/CAM materials.	Journal Ceramics-Silikáty	In vitro study
***16*** [22]	Fouda, A.M., Atta, O., Özcan, M., Stawarczyk, B., Glaum, R., Bourauel, C.	2023	An investigation on fatigue, fracture resistance, and color properties of aesthetic CAD/CAM monolithic ceramics.	Clinical Oral Investigations	In vitro study
***17*** [23]	Lu, Y., Dal Piva, A.M.O., Nedeljkovic, I., Tribst, J.P.M., Feilzer, A.J., Kleverlaan, C.J.	2023	Effect of glazing technique and firing on surface roughness and flexural strength of an advanced lithium disilicate.	Clinical Oral Investigations	In vitro study
***18*** [24]	Attar, E.A., Aldharrab, A., Ajaj, R.	2023	Flexural Strength Properties of Five Different Monolithic Computer-Aided Design/Computer-Aided Manufacturing Ceramic Materials: An In Vitro Study.	The Cureus Journal of Medical Science	In vitro study
***19*** [25]	Diken Türksayar, A.A., Demirel, M., Donmez, M.B.	2023	Optical properties, biaxial flexural strength, and reliability of new-generation lithium disilicate glass-ceramics after thermal cycling.	Journal of Prosthodontics	In vitro study
***20*** [26]	Freitas, J.S., Souza, L.F.B., Dellazzana, F.Z., Silva, T.M.R.D., Ribeiro, L., Pereira, G.K.R., May, L.G.	2023	Advanced lithium disilicate: A comparative evaluation of translucency and fatigue failure load to other ceramics for monolithic restorations.	Journal of the Mechanical Behavior of Biomedical Materials	In vitro study
***21*** [27]	Murillo-Gómez, F., Murillo-Alvarado, F., Vásquez-Sancho, F., Avendaño, E., Urcuyo, R.	2024	Effect of “fast”-crystallization and simultaneous glazing on physicochemical properties of lithium-disilicate CAD/CAM ceramic.	Journal of Dentistry	In vitro study
***22*** [28]	Zaniboni, J.F., Silva, A.S., Silva, A.M., Besegato, J.F., Muñoz-Chávez, O.F., de Campos, E.A.	2024	Microstructural and flexural strength of various CAD-CAM lithium disilicate ceramics.	Journal of Prosthodontics	In vitro study

**Table 3:** Specific data of the selected articles.

Material	Commercial Brand	Composition	Ceramic Group	E-Study	N	Young’s Modulus (GPa)	Flexural Strength (MPa)	Fracture Strength (k_ic_) (MPa·m^1/2^)	Vickers Hardness (GPa)	Roughness (µm)	Wear (mm^3^)	Translucency
**IPS e.max^®^ Press**	Ivoclar Vivadent (Amherst, NY, USA)	SiO_2_ (64.2%) Li_2_O (26%) K_2_O (2.5%) ZnO (1.47%) Al_2_O_3_ (1.30%) P_2_O_5_ (1.76%) Otros	**DSL**	1 [6]	*n* = 15 blocks	100.8		2.25 ± 0.17				
				2 [8]	*n* = 15 blocks	79.77 ± 9.76	249.59 ± 75.08					
				3 [9]	*n* = 40 discs		446 ± 81	1.03 ± 0.05	6.1 ± 0.12	Ra 0.01 ± 0.001 Rz 0.05 ± 0.002		
				4 [10]	*n* = 15 bars		303 ± 56	2.76 ± 0.4			−0.118 ± 0.013	
				5 [11]	*n* = 7 crowns			(Newton) 1706.01 ± 154.32 N				
				6 [12]	*n* = 20 blocks			2.50 ±0.31				
				7 [13]	*n* = 60 blocks		344.35 ± 65.94					
**IPS e.max^®^ CAD**	Ivoclar Vivadent	SiO_2_ (68.3%) Li_2_O (24.3%) K_2_O (2.42%) Al_2_O_3_ (1.97%) P_2_O_5_ (1.33%) Otros	**DSL**	1 [6]	*n* = 15 blocks	102.5		2.13 ± 0.05				
				2 [8]	*n* = 15 blocks	79.33 ± 17.39	364.64 ± 66.51					
				4 [10]	*n* = 60 blocks		345.74 ± 68					
				6 [12]	*n* = 20 blocks			1.79 ± 0.26				
				8 [14]	*n* = 30 bars	60.6 ± 1.64	348.33 ± 28.69	2.01 ± 0.13	5.45 ± 0.28			
				9 [15]	*n* = 30 discs		415 ± 26					26.0 ± 0.6
				10 [16]	*n* = 32 crowns			(Newtons) 2648 N				
				11 [17]	*n* = 8 crowns		648	(Newtons) 2529 ± 468.7 N		Ra: 4.4 ± 1.1 Rz: 33.2 ± 9.5		
				12 [18]	*n* = 10 discs		424.3 ± 52.26					27.82 ± 0.52
				13 [19]	*n* = 5 crowns			(Newtons) 1565.2 ± 89.7				
				14 [20]	*n* = 10 blocks		418.22 ± 53.98		5.46 ± 0.05			
				15 [21]	*n* = 20 discs		605 ± 104.3					
				16 [22]	*n* = 20 crowns			(Newtons) 1794 ± 288				15.6 ± 0.4
				17 [23]	*n* = 20 bars		370.6 ± 59.3			Ra: 0.17 ± 0.05 Rz: 1.23 ± 0.35		
				18 [24]	*n* = 10 blocks		372.68 ± 24.10					
				19 [25]	*n* = 10 discs		560.56 ± 48.17					20.33 ± 0.15
				20 [26]	*n* = 15 discs					Ra: 0.36 Rz: 2.38		28.27 ± 1.45
				21 [27]	*n* = 30 bars		427.48 ± 42.41					
				22 [28]	*n* = 15 blocks		371.26 ± 109.17					
**Initial™ LiSi Press**	GC (Houston, TX, USA)	SiO_2_ (66.8%) Li_2_O (24.3%) Al_2_O_3_ (2.93%) K_2_O (1.29%) Na_2_O (1.33%) Otros	**DSL**	1 [6]	*n* = 15 blocks	102.9		2.11 ± 0.10				
				2 [8]	*n* = 15 blocks	76.97 ± 7.20	203.54 ± 38.68					
				3 [9]	*n* = 40 discs		520 ± 100	1.02 ± 0.04	6.4 ± 0.03	Ra 0.01 ± 0.001 Rz 0.05 ± 0.002		
				4 [10]	*n* = 15 bars		251 ± 47	2.38 ± 0.4			−0.106 ± 0.013	
**Initial™ LiSi Block**	GC	SiO_2_ (68%) Li_2_O (22%) Al_2_O_3_ (2.08%) K_2_O (1.49%) Na_2_O (1.19%) Otros	**DSL**	1 [6]	*n* = 15 blocks	95.6		1.50 ± 0.04				
				16 [23]	*n* = 20 crowns			(Newtons) 1237 ± 263				18.17 ± 0.7
				19 [26]	*n* = 10 discs		458.50 ± 16.09					21.32 ± 1.77
**Vita Suprinity^®^**	Vita Zahnfabrik (Bad Säckingen, Germany)	SiO_2_ (54.7%) Li_2_O (34.09%) ZrO_2_ (4.52%) P_2_O_5_ (2.31%) K_2_O (1.14%) Al_2_O_3_ (1.13%) Otros	**ZLS**	1 [6]	*n* = 15 blocks	102.9		1.57 ± 0.04				
				8 [14]	*n* = 30 bars	70.44 ± 1.97	443.63 ± 38.90	2.31 ± 0.17	6.53 ± 0.56			
				9 [15]	*n* = 30 discs		510 ± 43					31.0 ± 1
				10 [16]	*n* = 32 crowns			(Newtons) 2923 N				
				12 [18]	*n* = 10 discs		549.4 ± 79.71					22.47 ± 0.8
				13 [19]	*n* = 5 crowns			(Newtons) 1742.9 ± 102.7				
				14 [20]	*n* = 10 blocks		281.23 ± 49.43		5.20 ± 0.15			
				15 [21]	*n* = 20 discs		330.7 ± 58.4					
				18 [25]	*n* = 10 blocks		428.48 ± 12.39					
				20 [27]	*n* = 15 discs					Ra: 0.10 Rz: 1.22		25.51 ± 0.54
**Celtra^®^ Press**	Dentsply (Woodbridge, ON, Canada)	Li_2_Si_2_O_5_ (58–65%) ZrO_2_ (10%) Óxidos de aluminio y de otros metales	**ZLS**	3 [9]	*n* = 40 discs		458 ± 113	0.74 ± 0.03	6.1 ± 0.05	Ra 0.01 ± 0.008 Rz 0.04 ± 0.007		
				4 [10]	*n* = 15 bars		320 ± 63	2.36 ± 0.4			−0.155 ± 0.034	
				5 [11]	*n* = 7 crowns			(Newtons) 1550.67 ± 196.71 N				
**Celtra^®^ Duo**	Dentsply	SiO_2_ (54.7%) Li_2_O (34.9%) ZrO_2_ (4.52%) P_2_O_5_ (2.31%) K_2_O (1.14%) Al_2_O_3_ (1.13%) Otros	**ZLS**	1 [6]	*n* = 15 blocks	107.6		1.51 ± 0.06				
				14 [20]	*n* = 10 blocks		246 ± 39.81		4.97 ± 0.09			
				16 [23]	*n* = 20 crowns			(Newtons) 1176 ± 323				16.5 ± 0.6
**CEREC Tessera™**	Dentsply	Li_2_Si_2_O_5_ (90.0%) Li_3_PO_4_ (5.0%) Li_0.5_Al_0.5_Si_2.5_O_6_ (virgilite): 5%	**ALD**	1 [6]	*n* = 15 blocks	103.1		1.45 ± 0.10				
				11 [17]	*n* = 8 crowns		>700	(Newtons) 2101.4 ± 752.6		Ra: 4.1 ± 1.6 Rz: 29 ± 14.2		
				12 [18]	*n* = 20 discs		463.22 ± 48.55					26.37 ± 0.86 24.91 ± 0.86
				17 [24]	*n* = 20 bars		313.6 ± 52.5			Ra: 0.07 ± 0.01 Rz: 1.46 ± 0.64		
				20 [27]	*n* = 15 discs					Ra: 0.04 Rz:0.66		28.14 ± 2.09
**Amber^®^ Mill**	HASS (Oxnard, CA, USA)	SiO_2_ (69.8%) Li_2_O (23%) K_2_O (1.77%) Al_2_O_3_ (1.68%) P_2_O_5_ (1.50%) Otros	**DSL**	1 [6]	*n* = 15 blocks	98.3		1.71 ± 0.04				
				19 [26]	*n* = 10 discs		514.08 ± 33.03					23.90 ± 1.02
**Amber^®^ Press**	HASS	SiO_2_ (62.9%) Li_2_O (28.1%) K_2_O (2.50%) ZnO (1.31%) Al_2_O_3_ (1.21%) P_2_O_5_ (1.47%) Otros	**DSL**	1 [6]	*n* = 15 blocks	105.5		2.29 ± 0.08				
				4 [19]	*n* = 15 bars		324 ± 43	2.86 ± 0.3			−0.117 ± 0.025	
**N!CE^®^**	Straumann AG (Basel, Switzerland)	SiO_2_ (63.2%) Li_2_O (22.8%) Al_2_O_3_ (6.22%) Na_2_O (2.49%) P_2_O_5_ (2.42%) CaO (1.51%) Otros	**DSL**	1 [6]	*n* = 15 blocks	91.7		1.53 ± 0.05				
**Obsidian^®^**	Glidewell (Newport Beach, CA, USA)	SiO_2_ (56.6%) Li_2_O (30%) K_2_O (2.66%) B_2_O_3_ (1.94%) Al_2_O_3_ (1.62%) P_2_O_5_ (1.12%) Otros	**DSL**	1 [6]	*n* = 15 blocks	100.04		1.84 ± 0.06				
**Livento Press**	Cendres + Metaux (Biel, Switzerland)	Li_2_Si_2_O_5_ (60–65%) SiO_2_ (55–65%) K_2_O_2_-Na_2_O (3–5%) Al_2_O_2_ (<1%) Otros	**DSL**	4 [19]	*n* = 15 bars		301 ± 22	2.67 ± 0.2			−0.114 ± 0.039	
**Rosetta SM**	HASS	Li_2_Si_2_O_5_ (60–65%) SiO_2_ (55–65%) Al_2_O_2_ (<1%) Otros	**DSL**	14 [20]	*n* = 10 blocks		369.59 ± 74.86		4.98 ± 0.38			
				22 [28]	*n* = 15 blocks		315.27 ± 94.17					

**Table 4:** Quality of the articles using the modified CONSORT scale for in vitro studies of dental materials. An asterisk (*) denotes compliance with the corresponding item.

*E-Studies*	1	2a	2b	3	4	5	6	7	8	9	10	11	12	13	14
*Lubauer, J., et al. [6]*	*	*	*	*	*	*					*				
*Al-Thobity, A.M., et al. [8]*	*	*	*	*	*	*					*	*	*		
*Hallmann, L., et al. [9]*	*	*	*	*	*	*					*				
*Stawarczyk, B., et al. [10]*	*	*	*	*	*	*					*	*	*		
*Salem, B.O., et al. [11]*	*	*	*	*	*	*					*				
*Alkadi, L., et al. [12]*	*	*	*	*	*	*					*		*		
*Fabian Fonzar, R., et al. [13]*	*	*	*	*	*	*					*				
*Elsaka, S.E., et al. [14]*	*	*	*	*	*	*					*		*		
*Sen, N., et al. [15]*	*	*	*	*	*	*					*				
*Sieper, K., et al. [16]*	*	*	*	*	*	*					*				
*Rosentritt, M., et al. [17]*	*	*	*	*	*	*					*		*		
*Demirel, M., et al. [18]*	*	*	*	*	*	*					*	*	*		
*Hamza, T.A., et al. [19]*	*	*	*	*	*	*					*	*	*		
*Corado, H.P.R., et al. [20]*	*	*	*	*	*	*					*				
*Shono, N., et al. [21]*	*	*	*	*	*	*					*	*	*		
*Fouda, A.M., et al. [22]*	*	*	*	*	*	*					*		*		
*Lu, Y., et al. [23]*	*	*	*	*	*	*					*		*		
*Attar, E.A., et al. [24]*	*	*	*	*	*	*					*		*		
*Diken Türksayar, A.A., et al. [25]*	*	*	*	*	*	*					*	*	*		
*Freitas, J.S., et al. [26]*	*	*	*	*	*	*					*		*		
*Murillo-Gómez, F., et al. [27]*	*	*	*	*	*	*					*	*	*		
*Zaniboni, J.F., et al. [28]*	*	*	*	*	*	*					*		*		

**Table 5:** Meta-analysis results.

Properties	Ceramic Group	Mean (IC 95%)	Q-Test for the Difference Between Groups	Independent Variables of the Meta-Regression
Young modulus	LDS	73.9 (61.7–86.2)	Q = 0.31 *p* < 0.577	No significance
	ZLS	70.4 (69.7–71.1)		
Fracture resistance	ALD	1.5 (1.4–1.5)	Q = 22.97 *p* < 0.05	No significance
	LDS	2 (1.8–2.2)		
	ZLS	1.7 (1.2–2.2)		
Flexural resistance	ALD	388.1 (241.5–534.7)	Q = 0.126 *p* = 0.939	Press technique (*p* < 0.05)
	LDS	384.7 (353–416.3)		
	ZLS	395.7 (343.9–447.5)		
Hardness	LDS	5.7 (5.2–6.2)	Q = 0.001 *p* = 0.979	Pressed technique (*p* < 0.05)
	ZLS	5.7 (5–6.4)		
Wear	LDS	−0.11 (−0.12–(−0.11))	Q = 19.15 *p* < 0.05	ZLS (*p* < 0.05)
	ZLS	−0.16 (−0.17–(−0.14))		
Roughness (Ra)	ALD	2.04 (−1.903–5.994)	Q = 5.345 *p* = 0.069	ALD (*p* < 0.05) Pressed technique (*p* < 0.05)
	LDS	0.015 (0.011–0.019)		
	ZLS	0.01 (0.008–0.012)		
Roughness (Rz)	ALD	14.8 (−12.2–41.7)	Q = 11.102 *p* < 0.05	ZLS (*p* < 0.05) Pressed technique (*p* < 0.05)
	LDS	0.05 (0.05–0.06)		
	ZLS	0.04 (0.038–0.042)		
Wear	LDS	−0.11 (−0.12–(−0.11))	Q = 0.001 *p* = 0.979	ZLS (*p* < 0.05)
	ZLS	−0.16 (−0.17–(−0.14))		
Translucency	ALD	27.2 (25.4–28.1)	Q = 4.576 *p* = 0.101	NS
	LDS	23.7 (20.7–26.6)		
	ZLS	23.9 (17.4–30.3)		

## Data Availability

No new data were created or analyzed in this study.

## Supplementary Materials

The following supporting information can be downloaded at: https://www.mdpi.com/article/10.3390/ma18122709/s1, The PRIMAS flow diagram from 2020 [7].
