# Supplementary material for: Analysis of Different Lithium Disilicate Ceramics According to Their Composition and Processing Technique—A Systematic Review and Meta-Analysis
Source: Materials (Basel). 2025 Jun 9;18(12):2709. doi: 10.3390/ma18122709 (PMC12194564; doi:10.3390/ma18122709)
Supplement: Supplementary file 1 [file materials-18-02709-s001.zip › materials-3636958-supplementary.pdf]

**PRISMA 2020 flow diagram for new systematic reviews which included searches of databases and registers only**

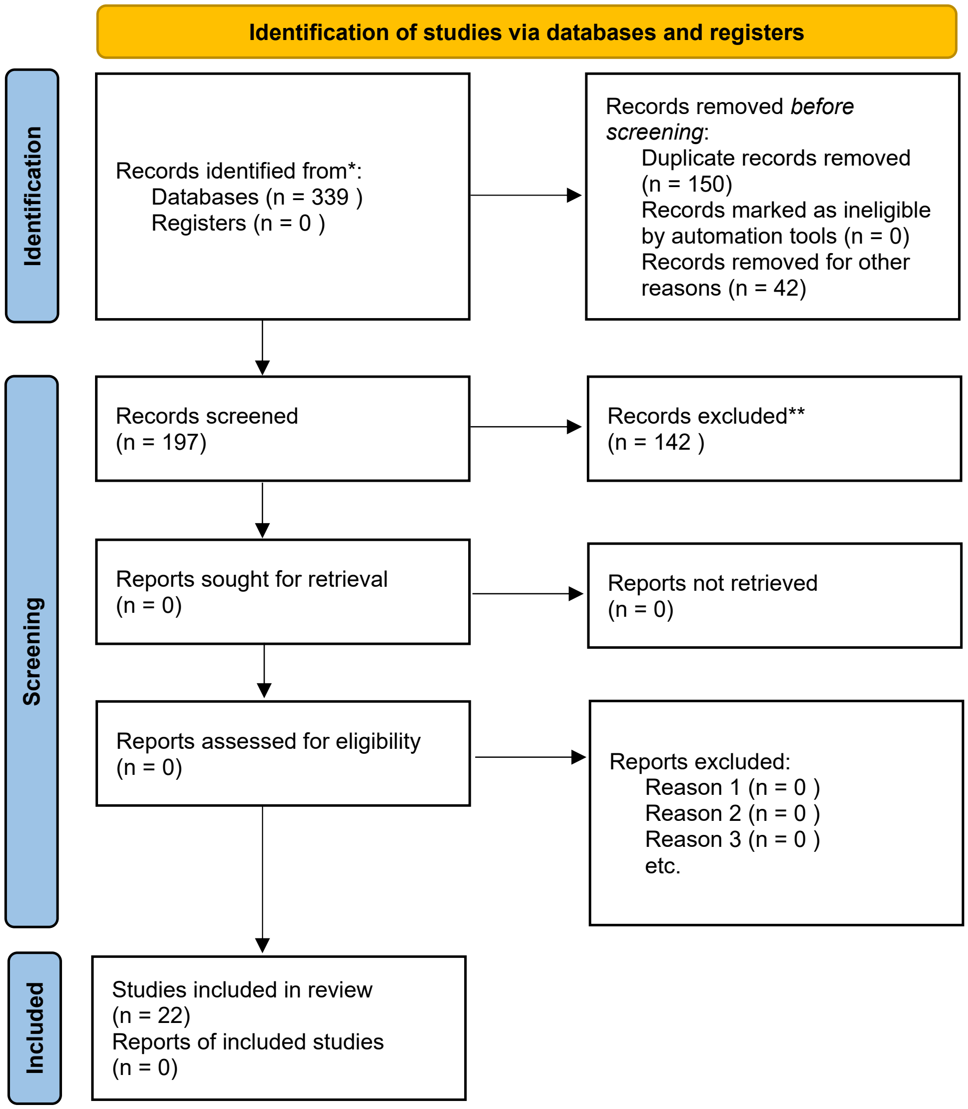


*Consider, if feasible to do so, reporting the number of records identified from each database or register searched (rather than the total number across all databases/registers).

**If automation tools were used, indicate how many records were excluded by a human and how many were excluded by automation tools.

Source: Page MJ, et al. BMJ 2021;372:n71. doi: 10.1136/bmj.n71.

This work is licensed under CC BY 4.0. To view a copy of this license, visit https://creativecommons.org/licenses/by/4.0/
